# Circulating miR-16-5p, miR-92a-3p and miR-451a are biomarkers of lung cancer in Tunisian patients

**DOI:** 10.1186/s12885-024-12181-1

**Published:** 2024-04-04

**Authors:** Alya Boutabba, Fadoua Missaoui, Akram Dlala, Hela Kamoun, Khalil Ben Salem, Amira Gabsi, Hadhemi Rejeb, Anne Letessier, Benoit Miotto, Raja Marrakchi

**Affiliations:** 1 Laboratory of genetics, immunology, and human pathologies, LR05ES05, Faculty of sciences, University of Tunis El-Manar - Campus Universitaire El-Manar, El-Manar Tunis, 2092 Tunisia; 2 Ibn Nafiss Pneumology Department, Abderrahmen Mami Hospital, Ariana, Tunisia; 3 Faculty of medicine, University Tunis El-Manar, Tunis, Tunisia; 4 Université Paris Cité, INSERM, U1016, CNRS, UMR8104, Institut Cochin, Paris, F-75014 France; 5 Team “Epigenetics, DNA replication and Cancer”, Institut Cochin, Paris, France

**Keywords:** Lung cancer, miRNA, Biomarker, Prognostic, Tunisian population, Diagnostic tools

## Abstract

Lung cancer is one of the most common type of cancer and, despite significant advances in screening and diagnosis approaches, a large proportion of patients at diagnosis still present advanced stages of the disease with distant metastasis and bad prognosis. Finding and validating biomarkers of lung cancer is therefore essential. Such studies are often conducted on European, American and Asian populations and the relevance of these biomarkers in other populations remains less clear. In that prospect, we investigated the expression level of seven microRNAs, chosen from the medical literature (miR-16-5p, miR-92a-3p, miR-103a-3p, miR-375-3p, miR-451a, miR-520-3p and miR-let-7e-5p), in the blood of Tunisian lung cancer patients, treated or not by chemotherapy, and healthy control individuals. We found that high expression levels of circulating miR-16-5p, miR-92a-3p and miR-451a in the plasma of untreated patients discriminate them from healthy control individuals. In addition, miR-16-5p and miR-451a expression levels are significantly reduced in the plasma of chemotherapy-treated patients compared to untreated patients. Our results confirmed previous work in other populations worldwide and provide further evidence that circulating miR-16-5p, miR-92a-3p and miR-451a potentially regulate key pathways involved in the initiation and progression of cancer.

**Supplementary Information:**

The online version contains supplementary material available at 10.1186/s12885-024-12181-1.

## Introduction

Lung cancer is the most frequent type of cancer in both sexes worldwide, representing a leading cause for cancer-related deaths [1, 2]. Despite advances in its treatment, lung cancer morbidity and mortality remain high. Early-stage lung cancer is characterized by very mild and unspecific symptoms and most patients already have local and distant invasion or metastasis when first diagnosed, which limits the effectiveness of treatments and the survival of patients [1, 2]. Many molecular biomarkers (i.e. mRNAs, proteins, non-coding RNAs…) have been validated for diagnosis, prognosis and prediction of therapeutic response in order to improve the prognosis of lung cancer patients. However, most of these studies have been carried out historically on European and North American populations, and they reproducibility in other populations around the world needs to be assessed.

The use of liquid biopsies is promising in that prospect since the measure of biomarkers (circulating tumour cells, mRNAs, proteins, non-coding RNAs…) in the peripheral blood of lung cancer patients can provide information onto the tumour (e.g. histologic subtype), the progression of the disease and the response to therapy [3]. Some cancer-associated circulating microRNAs (miRNAs) were reported to be present in the blood of lung cancer patients as compared to healthy individuals and some of these miRNAs, or combination of miRNAs, have promising clinical value for the classification and the prognosis of lung cancer patients [3–9].

miRNAs are short (18–24 nucleotides in length), stable, non-coding RNAs that regulate the stability and/or translation of their target messenger RNAs causing the deregulation of many genes important for cell proliferation, invasion, metastasis and apoptosis [10]. miRNAs can therefore exhibit oncogenic or tumour suppressive activities in cancer and several of these miRNAs have been identified as promising candidate biomarkers in lung cancer [4–8, 11–14]. Yet, data from European, American and Asian studies indicate significant variability across ethnicities that may be linked to different lifestyle, smoking habits, diet and environments [1, 2, 7, 14, 15]. This accentuates the need to further study miRNAs in diverse populations worldwide to be able to draw a clear view of miRNAs impact on lung cancer and as universal diagnostic tools.

In this study, we investigated the expression levels of seven miRNAs identified as biomarker of lung cancer in western populations in a cohort of Tunisian patients. We monitored by quantitative RT-PCR the expression levels of miR-16-5p, miR-92a-3p, miR-103a-3p, miR-375-3p, miR-451a, miR-520-3p and miR-let-7e-5p. These miRNAs were chosen based on the literature and their known deregulation in patients from different countries and ethnicities. miR-92a is expressed at higher levels in liquid biopsies of lung cancer patients compared to healthy control individuals [13, 16, 17]. miR-375 is expressed at higher levels in lung cancer tumours compared to normal lung tissue. Furthermore, it is differentially expressed in adenocarcinoma compared to squamous cell carcinoma, helping the molecular distinction between the two tumour subtypes [18–20]. miR-let-7e-5p is less expressed in lung cancer patient compared to healthy individuals [11, 14, 21]. miR-103a-3p expression is higher in plasma and tumour biopsies from patients with cisplatin resistant lung cancer tumours compared to patients with sensitive tumours [22]. miR-16-5p, miR-92a-3p and miR-451a are expressed at higher levels in plasma from North American lung cancer patients than control healthy individuals [13]. Finally, miR-520-3p is expressed at lower levels in lung cancer tumours compared to non-tumour tissues [23, 24].

Here, we monitored the expression of these seven miRNAs at the plasma level on 3 groups of individuals: untreated lung cancer patients, lung cancer patients treated with chemotherapy and healthy control individuals. We also monitored the expression of these miRNAs in peripheral blood mononuclear cells (PBMCs) from untreated lung cancer patients and healthy control individuals. We report differentially expressed miRNAs in plasma and PBMCs from Tunisian patients with treated and untreated lung cancer identifying biomarkers of lung cancer and chemotherapy treatment in this population. We further investigate the association of 3 circulating miRNAs (miR-16-5p, mir92a-3p and miR-451a), linked with lung cancer in our cohort, with their potential gene targets involved in lung tumourigenesis.

## Materials and methods

### Ethics statement

This study was approved by the local Medical Bioethics Committee at the Pasteur Institute of Tunis under the reference number 2015/04/E/FST. All individuals provided written informed consent to participate in this work. Healthy control individuals are under the age of 65 in accordance with the policies and regulations of the Tunisian Ministry of Health.

### Participants: blood samples and clinical data

Patients with lung cancer were recruited at the Abderrahmane MAMI hospital (Ariana, Tunisia): 44 patients were under chemotherapy treatment and were recruited from the oncology department between 2018 and 2023 (treated patients), and 29 did not receive any treatment and were recruited from Ibn Nafees medical department between 2020 and 2023 (untreated patients). Most patients were in an advanced stage of lung cancer (stage III and IV) and with non-small-cell lung cancer (NSCLC). Twenty-five control blood samples were collected through the military transfusion centre of Tunis at the same period from healthy volunteers with no history of malignant diseases. An overview of the groups, group size, median age, clinical information is presented in Table 1.

**Table 1:** Clinical characteristics of individuals recruited and of plasma and PBMCs analysed after quality control of samples

Variable	Cohort of patients recruited			Plasma samples analysed (after passing quality control)			PBMCs analysed (after passing quality control)	
	Healthy control individuals *n* = 25	Lung cancer patients		Healthy control individuals *n* = 24	Lung cancer patients		Healthy control individuals *n* = 17	Lung cancer patients untreated *n* = 29
		Untreated *n* = 29	Treated with chemotherapy *n* = 47		Untreated *n* = 29	Treated with chemotherapy *n* = 37		
**Gender**								
Male	21	25	39	21	25	33	17	25
Female	4	4	8	3	4	4	0	4
**Age**								
≥ 60	0	7	26	0	7	17	0	7
< 60	25	22	21	24	22	20	17	22
**Tumour subtype**								
NSCLC		29	46		29	36		29
SCLC		0	1		0	1		0
**TNM stage**								
Stage I		0	2		0	0		0
Stage III-IV		29	45		29	37		29
**Chemo-therapy regimen**								
Cisplatine + Gemzar®			18			14		
Cisplatine + Navelbine®			23			20		
Cisplatine + Taxol®			6			3		

*NSCLC* Non-small-cell lung cancer, *SCLC* Small-cell lung cancer, *TNM* Tumour, Node, Metastasis

### Separation of blood cells on a Ficoll density gradient

8 mL of peripheral vein whole blood were obtained from each subject, and the peripheral blood mononuclear cells (PBMCs) were isolated using density gradient centrifugation with Ficoll solution according to operating instructions (Eurobio, catalog number CMSMSL01-01). The peripheral vein whole blood was diluted with PBS (×1) in 1:1 ratio, and the mixture was transferred into a 50 mL centrifugal tube pre-filled with 8 mL Ficoll (Eurobio, catalog number CMSMSL01-01). The PBMCs were carefully collected in the middle layer of the tube following centrifugation for 30 min (500 g). Subsequently, the PBMCs were washed and centrifuged with PBS 1X for 5 min at 350 g and re-suspended in 10% DMSO / 90% foetal calf serum and cryopreserved at -80 °C. At the same time, the plasma layer was collected, centrifuged to remove cellular debris and stored at -80 °C until use. Haemolysis was quantified by assessing the presence of cell free haemoglobin by measuring the absorbance at 414 nm with a microplate spectrophotometer (Agilent BioTek Epoch with Gen5 data analysis software). Samples with signs of haemolysis (OD > 0.3) were removed from the analysis (Supplementary Material 1).

### RNA extraction from PBMC and plasma samples, and reverse transcription

Total RNAs were prepared using TriReagent protocol (Sigma-Aldrich, catalogue number T9424) and followed by a standard chloroform purification procedure from PBMC and plasma. Briefly, 200 µL of plasma was mixed with 800 µL of TriReagent. PBMCs were centrifuged and the cell pellet resuspended in 1 mL of TriReagent. Mixes were then vortexed 15 s and incubated for 10 min at room temperature. 200 µL of chloroform was added to each mix, vortexed for 15 s, incubated for 10 min at room temperature and then centrifuged for 30 min at 4 °C. Supernatants were transferred in clean tubes, 700 µL of isopropanol added, as well as glycogen as carrier (1 µg/µL final concentration; Thermo Fisher Scientific, catalogue number R0561) and 50 µL of 3 M sodium acetate. The tubes were incubated overnight in a -80 °C freezer, centrifuged at 4 °C for 30 min and the RNA pellet washed with ice-cold ethanol 70%, dried and resuspended in 50 µL DNAse- and RNAse-free water. RNA quality and concentration were evaluated using a Nanodrop™ instrument (Thermo Fisher Scientific).

MiRNAs were reverse transcribed using miRCURY® LNA® RT kit according to manufacturer recommendations (QIAGEN, catalogue number 339,340). RNA samples were diluted to 5 ng/µL using nuclease-free water (Invitrogen, catalogue number 10977-035) and the reverse transcription reactions prepared on ice. We mixed 2 µL 5x miRCURY SYBR® Green RT Reaction Buffer (QIAGEN), 5 µL nuclease-free water (Invitrogen, catalogue number 10977-035), 1 µL 10x miRCURY RT Enzyme Mix (QIAGEN), 2 µL Template RNA (5 ng/µL) for a total reaction volume of 10 µL. The reactions were incubated for 60 min at 42 °C and then incubated for 5 min at 95 °C to heat inactivate the reverse transcriptase, and immediately cool to 4 °C.

### Real-time quantitative PCR analysis

Reverse transcribed miRNAs were analysed by real-time PCR on a LightCycler® 480 system (Roche) available at the GENOM’IC platform at Institut Cochin (INSERM U1016, Paris, France). Relative miRNA expression levels were determined on samples run in duplicate with the miRCURY® LNA® SYBR® Green PCR Kit (QIAGEN, catalogue number 339,345) using the 2-Cq method and a set of 3 endogenous noncoding RNAs (U6 snRNA, SNORD48 and miR-191-5p) for reference.

The miRCURY® LNA® miRNA PCR assay primers are commercially available (QIAGEN, catalog number 339,306) with the following GeneGlobe IDs: miR-let-7e-5p (YP00205711), miR-16-5p (YP00205702), miR-92a-3p (YP00204258), miR-103a-3p (YP00204063), miR-191-5p (YP00204306), miR-375-3p (YP00204362), miR-451a (YP02119305), miR-520-3p (YP00204074), SNORD48 (YP00203903) and U6 snRNA (YP02119494).

### Receiver operating characteristic (ROC) curves

Area under the ROC curve (AUC) was performed using GraphPad Prism (GraphPad Software). Analyses were performed by entering ∆Cp values for the different miRNAs from healthy control individuals and lung cancer patients (untreated). The sensitivity (%) was plotted as a function of the false-positive rate (calculated as 100% - specificity%) for the different cut-offs.

### Prediction of biological processes regulated by miRNAs

The miRTarBase 9.0 was interrogated on April 9, 2023 to list experimentally validated target genes of miR-16-5p, miR-451a and miR-92a-3p [25]. These lists of genes were intersected with the web-based Venny 2.1 interface (Oliveros, J.C., 2007, VENNY, https://bioinfogp.cnb.csic.es/tools/venny/) to identify common and specific target genes. These lists of genes were functionally annotated on April 10, 2023 using the g: Profiler interface with the g: GOSt function for enrichment analysis with default parameters [26].

### Statistical analysis

A Mann-Whitney U test was used to compare the different groups. *P*-value *P* < 0.05 was considered significant and the significance indicated as follow: n.s., not significant; *, *P* < 0.05; **, *P* < 0.01; ***, *P* < 0.001 and ****, *P* < 0.0001.

## Results

### miRNAs expression in the plasma of lung cancer patients

We monitored the expression level of seven miRNAs (miR-16-5p, miR-92a-3p, miR-103a-3p, miR-375-3p, miR-451a, miR-520-3p and miR-let-7e-5p) chosen according to information known in the literature [13, 16–24]. Expression was monitored at the plasma level in three groups: 29 untreated lung cancer patients, 37 (chemotherapy-)treated lung cancer patients and 24 healthy control individuals for whom no sign of haemolysis was detected in plasma (Supplementary Material 1) and a high-quality RNA preparation was obtained. Expression was normalised to the ubiquitous expression of three endogenous small RNAs (miR-191-5p, SNORD48 and U6 snRNA) and then compared between groups.

### Untreated lung cancer patients versus healthy control individuals

**Fig. 1:**
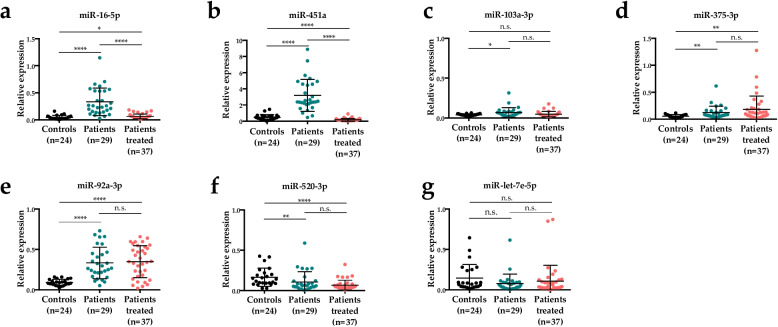
Expression level of miRNAs in the plasma of healthy control individuals (Controls), lung cancer patients without chemotherapy (Patients) and with chemotherapy (Patients treated). **a** Expression of miR-16-5p. **b** Expression of miR-451a. **c** Expression of miR-103a-3p. **d** Expression of miR-375-3p. **e** Expression of miR-92a-3p. **f** Expression of miR-520-3p. **g** Expression of miR-let-7e-5p. Mann-Whitney U test for comparison between groups; *P*-value significance: n.s., not significant; *, *P* < 0.05; **, *P* < 0.01; ***, *P* < 0.001 and ****, *P* < 0.0001

We observed that the expression level of miR-16-5p, miR-92a-3p, miR-103a-3p, miR-375-3p and miR-451a is significantly higher in untreated lung cancer patients compared to healthy control individuals (Fig. 1a-e). The level of expression of miR-520-3p is significantly lower in untreated lung cancer patients compared to healthy control individuals (Fig. 1f). The level of expression of miR-let-7e-5p is not different between untreated lung cancer patients and healthy control individuals (Fig. 1g). Fold-changes (FC) in expression are rather modest (FC < 2) for most miRNAs, except for miR-451a (FC = 5.67), miR-16-5p (FC = 6.55) and miR-92a-3p (FC = 2.65), which are expressed at higher levels in patients.

**Fig. 2:**
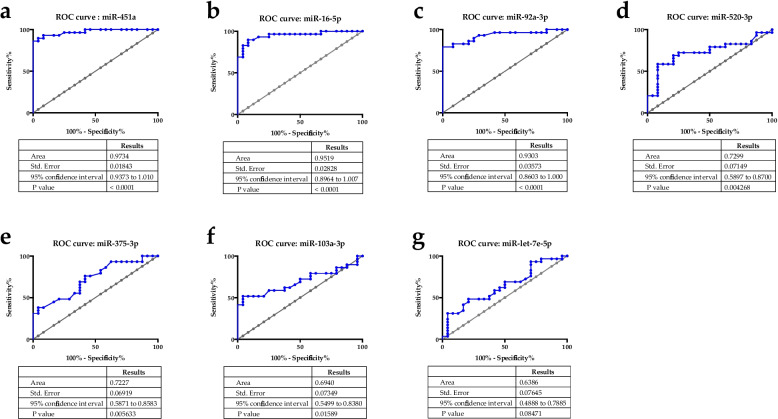
Area under the ROC curve (AUC) analyses, showing both sensitivity and specificity, for each miRNAs (untreated lung cancer patient/ healthy control individuals). The diagonal grey line reflects the performance of a diagnostic test that is no better than chance. Table under each graph summarizes statistics. **a** miR-451a. **b** miR-16-5p. **c** miR-92a-3p. **d** miR-520-3p. **e** miR-375-3p. **f** miR-103a-3p. **g** miR-let-7e-5p

To further explore the clinical significance of these miRNAs, we produced ROC curves between healthy control individuals and untreated lung cancer patients (Fig. 2). ROC curves show that miR-451a (AUC = 0.973), miR-16-5p (AUC = 0.951) and miR-92a-3p (AUC = 0.93) distinguish untreated lung cancer patients from healthy control individuals (AUC > 0.8) (Fig. 2a-c). These 3 miRNAs are thus promising miRNAs to help diagnose advanced lung cancer through blood analysis in the Tunisian population. The expression of the other miRNAs studied does not significantly discriminate the two groups (Fig. 2d-g).

We further considered the age disparity between the two groups (see Table 1). To determine whether age is a confounding factor, we took advantages on a previous study that identified age-associated microRNAs in peripheral blood [27]. This study revealed that miR-16-5p, miR-451a, miR-let7e-5p, and miR-92a-3p are under-expressed in the plasma of older (66 ± 9 years old) compared to younger (46 ± 9 years old) healthy individuals [27]. In our study, these miRNAs (miR-16-5p, miR-92a-3p and miR-451a) are over-expressed in plasma from cancer patients while miR-let7e-5p is not differentially expressed. We thus concluded that, despite the age difference between groups, there is no confounding effect of age in our analysis.

**Fig. 3:**
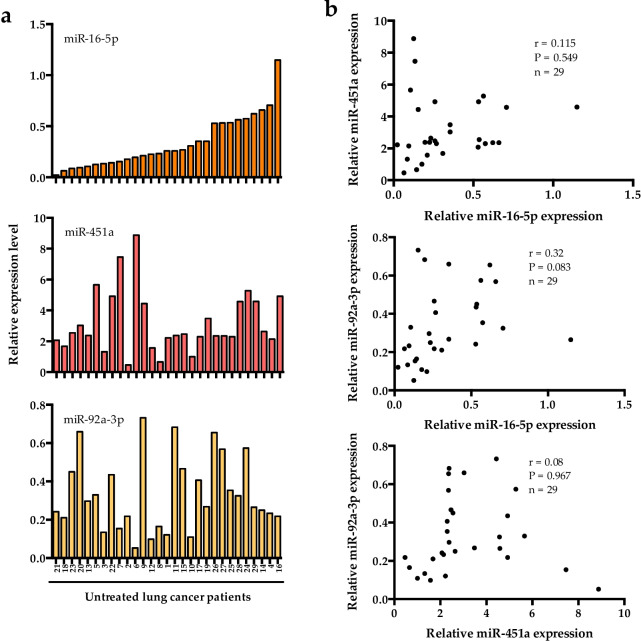
Correlation between miR-16-5p, miR-451a and miR-92a-3p expression levels in the plasma of untreated lung cancer patients. **a** Graph depicting miR-16-5p (top), miR-451a (middle) and miR-92a-3p (bottom) expression levels in the plasma of untreated lung cancer patients. Patients were ranked according to miR-16-5p expression level. **b** Graph showing pair-wise correlation between miR-16-5p and miR-451a expression (top), miR-16-5p and miR-92a-3p expression (middle) and miR-92a-3p and miR-451a expression (bottom). Pearson correlation (r), *P*-value (P) and number of untreated lung cancer patients analysed (n) are indicated

We then investigated whether the expression of miR-451a, miR-16-5p and miR-92a-3p was co-regulated in untreated cancer patients. The three miRNAs are highly expressed compared to the other miRNAs monitored (Fig. 1), still expression levels are not correlated in plasma samples from patients (Fig. 3). This result indicates that miR-451a, miR-16-5p and miR-92a-3p are probably not co-regulated.

### Untreated vs. treated lung cancer patients

We observed that the expression level of miR-16-5p and miR-451a is significantly lower in treated lung cancer patients compared to untreated patients (Fig. 1a, b). All other miRNAs (miR-92a-3p, miR-103a-3p, miR-375-3p, miR-520-3p and miR-let-7e-5p) are expressed at comparable levels between these two groups of individuals (Fig. 1c-g). Levels of miR-16-5p and miR-451a are thus affected by chemotherapy in lung cancer patients in the Tunisian population.

### Treated lung cancer patients versus healthy control individuals

miR-16-5p, miR-92a-3p and miR-375-3p expression is higher in treated patients compared to healthy control individuals (FC > 2; *P* < 0.05) while miR-451a and miR-520-3p expression is lower (Fig. 1). Levels of expression of several of the miRNAs we studied are thus regulated by chemotherapy regimen.

### miRNAs expression in PBMCs of lung cancer patients

**Fig. 4:**
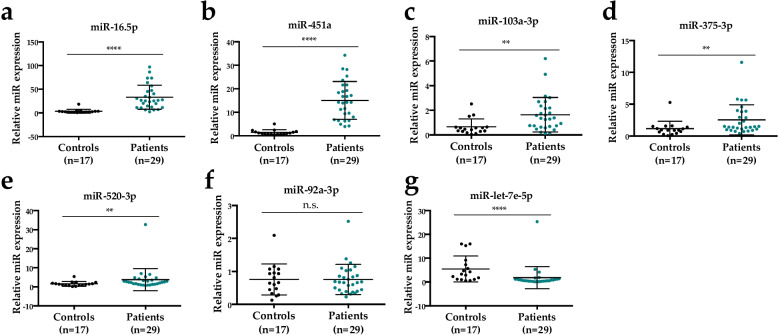
Expression level of miRNAs in PBMCs of healthy control individuals and lung cancer patients without chemotherapy. **a** Expression of miR-16-5p. **b** Expression of miR-451a. **c** Expression of miR-103a-3p. **d** Expression of miR-375-3p. **e** Expression of miR-520-3p. **f** Expression of miR-92a-3p. **g** Expression of miR-let-7e-5p. Mann-Whitney U test for comparison between groups; *P*-value significance: n.s., not significant; **, *P* < 0.01; ***, *P* < 0.001 and ****, *P* < 0.0001

To further validate our results, we investigated the expression of the seven miRNAs in PBMCs. We monitored miRNAs expression in PBMCs of 29 untreated lung cancer patients and 17 healthy control individuals by real-time qPCR. The expression levels of miR-16-5p, miR-103a-3p, miR-375-3p, miR-451a and miR-520-3p are significantly higher in PBMCs from lung cancer patients compared to healthy controls individuals (Fig. 4a-e). On the other hand, the level of expression of miR-92a-3p is similar between the two groups (Fig. 4f). The expression level of miR-let-7e-5p is significantly lower in PBMCs from lung cancer patients compared to healthy controls individuals (Fig. 4g).

We then compared the data in plasma and PBMCs of untreated lung cancer patients. We observed that data and conclusions drawn from the different blood sample types (plasma vs. PBMCs) are similar for all miRNAs but miR-92a-3p, miR-520-3p and miR-let-7e-5p. miR-92a-3p is highly expressed in the plasma of lung cancer patients compared to healthy control individuals (FC > 2), while its expression level is not different in PBMCs between the lung cancer patients compared to healthy control individuals. miR-520-3p is expressed at lower levels in the plasma of lung cancer patients compared to healthy control individuals, while its expression level is higher in PBMCs of cancer patients. miR-let-7e-5p is expressed at lower levels in PBMCs from lung cancer patients compared to healthy control individuals, while its expression level is not different in plasma samples between the two groups. This indicates that among the 3 miRNAs that are discriminating lung cancer patients from healthy control individuals, miR-92a-3p expression is more informative in plasma-based assays, while expression of miR-451a and miR-16-5p can be monitored both in plasma and PBMCs.

### Prediction of miRNAs regulation on biological processes

**Fig. 5:**
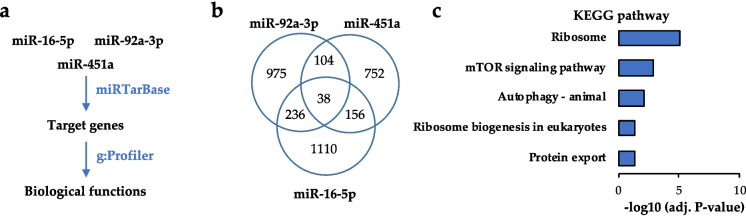
Functional annotation of miRNA target genes. **a** Identification of miRNA target genes and functional analysis. **b** Venn diagram showing common and specific target genes between miR-451a, miR-16-5p and miR-92a-3p. **c** Functional annotation (KEGG pathway) of the set of common genes

In order to understand the potential biology processes regulated by miR-16-5p, miR-92a-3p and miR-451a we identified experimentally validated target genes of these miRNAs using the miRTarBase [25]. We then intersected the lists of target genes and annotated the common set of target genes. We observed that cell-growth related pathways were enriched, including ribosome (i.e., mRNA translation), mTOR signalling and autophagy (Fig. 5). The PI3K/AKT/mTOR pathway is frequently altered in lung cancer and it is assumed that aberrant activation of this pathway contributes to tumour growth, tissue invasion, metastasis and drug resistance [28]. Autophagy and mRNA translation are central cellular processes. Reports show that they contribute to cancer progression and the resistance to chemotherapies in lung and other cancer types [29, 30]. This suggests that miR-451a, miR-16-5p and miR-92a-3p may play central functions on cell growth and cancer-related pathways in lung cancer.

## Discussion

### miRNAs expression in Tunisian lung cancer patients

Monitoring miRNAs expression levels in the plasma of lung cancer patients holds great promise for diagnosing the disease [4–8, 11–14, 16, 20, 31]. However, it is still needed to validate the robustness of the findings in different populations. We therefore studied the expression of seven miRNAs well known for their diagnostic value in the Western, Indian and/or Asian population in a cohort of Tunisian patients. There is currently very limited information regarding biomarkers and prognostic factors for lung cancer survival in the Tunisian population [32–35]. Our study is therefore not only corroborating previous studies and findings in the field, but it also identifies significant differences with studies in the Western, Indian and Asian population.

miR-16-5p, miR-92a-3p and miR-451a are very highly expressed in the plasma of Tunisian lung cancer patients compared to other circulating miRNAs we monitored. This is also observed in a more comprehensive manner in previous studies in the North American population [13]. In addition, the expression level of these miRNAs is higher in lung cancer patients than in healthy control individuals and ROC curve analysis indicate that the expression level of each miRNA can discriminate untreated lung cancer patients from healthy control individuals with high selectivity and sensitivity. This is consistent with previous studies reporting that miR-16-5p, miR-92a-3p and miR-451a are up-regulated in lung cancer patients from different countries [6, 13, 16, 17]. Importantly, our study interrogated untreated patients with advanced stage of the disease (stage III and IV) while other studies were also testing early stage of the disease (Stage I). This suggest that miR-16-5p, miR-92a-3p and miR-451a expression might be associated with the presence of lung cancer rather than a specific stage of the disease. It would thus be interesting to carry out a study in the Tunisian population to address this point by monitoring earlier stages of the disease and a larger cohort of patients. Analysis of a larger cohort would also provide an opportunity to address the question of whether certain factors have confounding effects. In our analysis, we concluded, on the basis of a previously published analysis of age-associated microRNAs, that despite the age difference between our groups, age did not introduce a confounding effect in our analysis. It would be interesting to test this further with a perfectly age-matched group of patients, and to also study other potential factors such as sex, diet and smoking habits.

We observed that miR-92a-3p expression is solely highly expressed in the plasma, but not in PBMCs, of untreated lung cancer patients suggesting that monitoring miR-92a-3p in the plasma might be highly informative to diagnose Tunisian lung cancer patients. In contrast, miR-92a-3p remains high in the plasma of patients treated with chemotherapy while the levels of miR-16-5p and miR-451a are almost indistinguishable from levels monitored in the plasma of healthy control individuals. This finding indicates that miR-92a-3p expression is not responsive to the chemotherapy and that miR-16-5p and miR-451a might be promising to help diagnose lung cancer and assess chemotherapy in the Tunisian population. It is reported that miR-92a-3p expression is lower in patients compared to healthy individuals in India, while it is higher in patients compared to controls in Western populations [14, 16, 17]. We observed that miR-92a-3p is higher in the plasma of Tunisian patients compared to healthy control individuals. This finding suggests that circulating miR-92a-3p expression level might depend partly on environmental conditions and/or genetic features shared by Tunisian and Western populations.

All other miRNAs studied were only modestly mys-regulated in patients compared to healthy control individuals (Fold change, FC < 2). This might render their use in the clinic more challenging. We observed that miR-let-7e-5p expression is lower in PBMCs of untreated lung cancer patients compared to healthy control individuals. This is consistent with previous work showing that this miRNA is down-regulated in lung cancer patients from India and other countries [11, 14, 21]. We observed that miR-375p expression is slightly higher in the plasma and PBMCs of Tunisian patients compared to healthy control individuals while its expression is lower in patients in other studies worldwide [18–20]. This suggests a relationship between miR-375p expression and environmental factors. Nonetheless, it is also important to note that miR-375 was found unsuitable as a biomarker in the Indian population [14]. Our data suggest that many miRNAs that are used in the clinic worldwide, for diagnosis and prognosis, may not be suitable for the diagnosis of Tunisian patients.

Our study reports the expression level of 7 miRNAs in the plasma and PBMCs of Tunisian lung cancer patients. Three of these miRNAs, namely miR-16-5p, miR-92a-3p and miR-451a distinguish untreated lung cancer patients from healthy control individuals, and our bioinformatics investigation predicts these miRNAs are important regulators of multiple pathways involved in cancer initiation and progression. In addition, we observed that miR-16-5p and miR-451a expression levels are reduced in cancer patients upon chemotherapy in the Tunisian population. The findings, although highly significant, and reproducing previous results in Western and Asian populations, will need further validation in other cohorts and with higher numbers of patients. Still, our study identified miR-16-5p, miR-92a-3p and miR-451a as promising markers of lung cancer in the Tunisian population.

### Supplementary Information

**Supplementary Material 1.**

## Data Availability

Raw data are available upon reasonable request from Benoit Miotto, benoit.miotto@inserm.fr.
