# Supplementary material for: Circulating miR-16-5p, miR-92a-3p and miR-451a are biomarkers of lung cancer in Tunisian patients
Source: BMC Cancer. 2024 Apr 4;24:417. doi: 10.1186/s12885-024-12181-1 (PMC10996140; doi:10.1186/s12885-024-12181-1)

Circulating miR-16-5p, miR-92a-3p and miR-451a are biomarkers of lung cancer in Tunisian patients

Alya Boutabba and colleagues.

Supplementary Material 1

Absorbance values at optical density 414 nm for haemoglobin in plasma samples. All samples included in our analysis had an optical density between 0.02 and 0.3, while samples showing signs of haemolysis (optical density > 0.3) were not considered.

H, Healthy control individuals; PNT, untreated lung cancer patient; PTC, lung cancer patient treated with chemotherapy; PBS, phosphate buffer saline (Gibco, reference: 14190-169); Water, UltraPure™ distilled water (Invitrogen, reference: 10997-035).

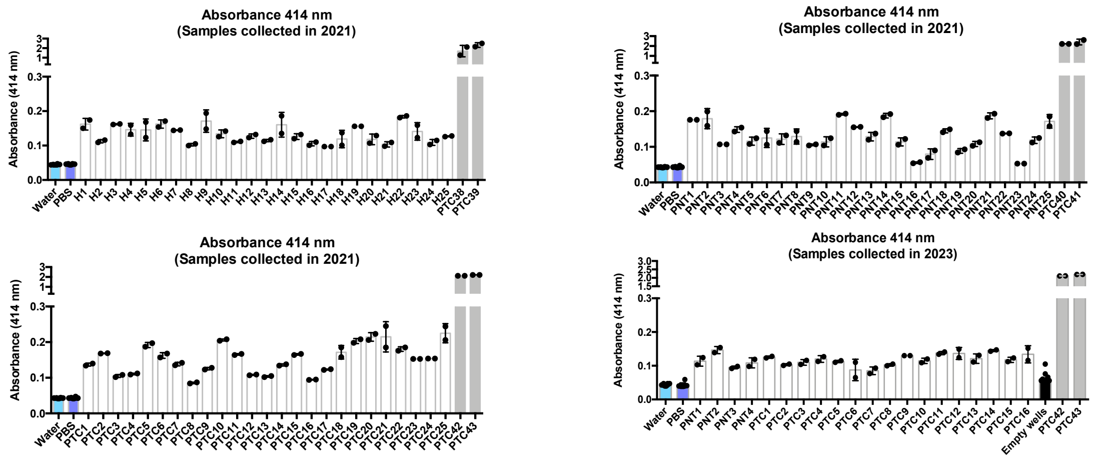

Supplement: Supplementary file 1 — Supplementary Material 1. [file 12885_2024_12181_MOESM1_ESM.pdf]
